# Misfit Layer Compounds and Ferecrystals: Model Systems for Thermoelectric Nanocomposites

**DOI:** 10.3390/ma8042000

**Published:** 2015-04-22

**Authors:** Devin R. Merrill, Daniel B. Moore, Sage R. Bauers, Matthias Falmbigl, David C. Johnson

**Affiliations:** Department of Chemistry and Materials Science Institute, University of Oregon, 1253 University of Oregon, Eugene, OR 97403, USA; E-Mails: devinm@uoregon.edu (D.R.M.); dan.b.moore@gmail.com (D.B.M.); bauers@uoregon.edu (S.R.B.); falmbigl@uoregon.edu (M.F.)

**Keywords:** thermoelectric materials, misfit layer compound, ferecrystal, electrical transport

## Abstract

A basic summary of thermoelectric principles is presented in a historical context, following the evolution of the field from initial discovery to modern day high-*zT* materials. A specific focus is placed on nanocomposite materials as a means to solve the challenges presented by the contradictory material requirements necessary for efficient thermal energy harvest. Misfit layer compounds are highlighted as an example of a highly ordered anisotropic nanocomposite system. Their layered structure provides the opportunity to use multiple constituents for improved thermoelectric performance, through both enhanced phonon scattering at interfaces and through electronic interactions between the constituents. Recently, a class of metastable, turbostratically-disordered misfit layer compounds has been synthesized using a kinetically controlled approach with low reaction temperatures. The kinetically stabilized structures can be prepared with a variety of constituent ratios and layering schemes, providing an avenue to systematically understand structure-function relationships not possible in the thermodynamic compounds. We summarize the work that has been done to date on these materials. The observed turbostratic disorder has been shown to result in extremely low cross plane thermal conductivity and in plane thermal conductivities that are also very small, suggesting the structural motif could be attractive as thermoelectric materials if the power factor could be improved. The first 10 compounds in the [(PbSe)_1+δ_]_m_(TiSe_2_)_n_ family (m, n ≤ 3) are reported as a case study. As n increases, the magnitude of the Seebeck coefficient is significantly increased without a simultaneous decrease in the in-plane electrical conductivity, resulting in an improved thermoelectric power factor.

## 1. Introduction

Thermoelectric research dates back to the 1820s when Seebeck discovered that a current flows in a closed circuit made of two different metals when the two junctions between the metals are at different temperatures [1]. Peltier discovered the reverse effect a decade later, setting the stage for both heating and cooling thermoelectric modules [2]. Initial devices were based on metal thermocouple junctions. The first significant breakthrough to improve performance was by Ioffe in the 1930s, who discovered that heavily doped semiconductors were more efficient than metals [3]. The ability to make them *n* doped and *p* doped enabled the development of useful thermoelectric modules. Later Ioffe introduced the concept of the figure of merit for thermoelectric materials [4]. A material’s dimensionless figure of merit is given by,

$$zT=\;\frac{{\text{α}}^{2}\text{σ}T}{\text{κ}}$$

where *T* is the temperature, α is the Seebeck coefficient, σ the electrical conductivity and κ the total thermal conductivity. κ is the sum of the electrical thermal conductivity, κ_e_, and the lattice thermal conductivity, κ_l_. α, σ and κ are all temperature dependent. To obtain high values of *zT*, the goal is to maximize the numerator (power factor) and minimize the denominator (total thermal conductivity) simultaneously to increase the *zT*. This requires decoupling of the above three key transport related properties in solids—α, σ and κ_e_. As discussed by Spaldin, these are “contraindicated” properties [5]. The electrical conductivity and Seebeck coefficient vary in opposite directions with the density of states at the Fermi level. The electrical and the electrical thermal conductivity vary together, resulting in the Wiedemann-Franz relationship [6]. The interrelationship between these properties presents one of the grand challenges in materials chemistry and physics—engineering new materials to obtain “contraindicated” properties. Three separate challenges need to be met. One needs to design new materials in which the scattering mechanisms and band structure can be altered to allow simultaneous increases in electrical conductivity and Seebeck coefficient, or at least to minimize the trade-off between them. One also needs to design chemical structures in which phonons and electrons move independently so as to minimize lattice thermal conductivity without reducing electron mobility. Third, one needs to be able to synthesize the designed materials to determine if the design criteria are correct and result in the targeted compounds having the expected properties.

## 2. Historical Context

### 2.1. Early Thermoelectric Materials Research

Ioffe and others prompted initial thermoelectric materials research in the 1950s based on several design concepts [7,8]. This decade and the 1960s focused on preparing and characterizing narrow band gap semiconductors and on making solid solutions to reduce the lattice thermal conductivity via mass difference (or alloy) scattering. There was a focus on using heavy elements from the right hand side the periodic table with small electronegativity differences under the assumption that this would result in high carrier mobility. These efforts resulted in finding thermoelectric materials such as bismuth telluride, with *zT* values around 1, which still are the dominant materials in commercial thermoelectric devices today. Thermoelectric materials research activity subsequently declined during the 1970s and 1980s, being limited in part by the lack of new semiconducting materials to be explored and also by the lack of new strategies that might significantly increase *zT*.

### 2.2. Improving Materials through Minimizing Lattice Thermal Conductivity

Starting in the mid-1990s, two new ideas inspired a tremendous surge of interest in the search for new thermoelectric materials with enhanced performance. In 1993, Hicks and Dresselhaus proposed that it might be possible to increase *zT* of certain materials by preparing them as part of quantum-well superlattice structures [9,10]. The idea was that the lower dimension of a two dimensional slab would introduce sharp features in the density of states that could result in an increased power factor. Two years later, Slack proposed the concept of a “phonon-glass electron-crystal” for designing efficient thermoelectric materials [11]. The idea was to lower the lattice thermal conductivity, which is independent of electrical transport properties, without degrading the electrical properties. The lowest thermal conductivities are commonly found in glasses due to the short mean free paths of phonons, hence the phonon glass in Slack’s concept. High *zT* values also require maximizing the numerator, which is commonly referred to as the power factor. The power factor is typically largest in materials with large mobility values, which are found in single crystals—the electron crystal part of Slack’s concept. Importantly, Slack suggested several types of structures where “phonon-glass electron-crystal” behavior might be found. These centered around structures that contain large vacancies where dopant atoms could be incorporated without disturbing the electronic crystal component. The concept was that dopant atoms would “rattle” around in the large cavity at operating temperatures, acting as an Einstein-like scatterer of phonons. The atoms forming the valence band would be matrix atoms, only weakly coupled to the dopant atoms, and therefore the compounds would maintain their large mobility values.

With these ideas introduced, the experimentalists quickly found very promising initial systems to explore. While there were several very promising early reports of extraordinarily high *zT* values from layered systems prepared to test the prediction of Hicks and Dresselhaus, the main cause of increased performance was a significant reduction in lattice thermal conductivity rather than an increase in the power factor [12,13]. Unfortunately, other research groups have not been able to reproduce the exceptionally high *zT* values in these initial reports [14]. The “phonon-glass electron-crystal” concept, on the other hand, has produced two major classes of materials, skutterudites [15] and clathrates [16], that now have compounds that rank in the top 10 of the highest *zT* values reported.

The renewed research efforts have also resulted in several other proposed mechanisms to increase *zT* and have been used as foundations to search for exceptional *zT* performance. It was noted that compounds with complex unit cells often have very low lattice thermal conductivities, and a very promising new thermoelectric material Yb_14_MnSb_11_ was discovered by Kauzlarich in 2007 [17,18]. Lee and later Morelli both showed that compounds near phase transitions can have very low lattice thermal conductivity and reasonable *zT* values [19,20]. Kanatzidis and others have shown that nanocomposites are another way to achieve exceptionally high *zT* values, with the inclusions of A in B resulting in very low lattice thermal conductivity and hints that the ability to control carrier concentration in part through charge transfer between the matrix and the inclusion and selective scattering of carriers might result in higher power factors [21].

To summarize the efforts in the last 20 years, there are now quite a few avenues to reduce lattice thermal conductivity without seriously impacting the power factor. This has led to several materials reported by more than one research group having *zT* values of ~1.5. There have even been reports of thermal conductivity values well below the previously assumed “predicted minimum thermal conductivity” as a result of efforts to optimize thermoelectric performance.

### 2.3. Enhancing Power Factors through Nanocomposites and Band Engineering

**Figure 1:**
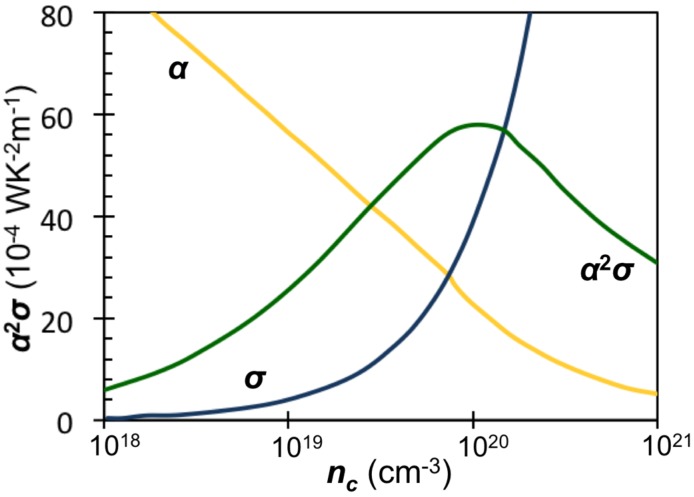
General relationship between Seebeck coefficient (α), conductivity (σ), and power factor (α^2^σ) as a function of carrier concentration (*n_c_*). Curves generated based on Bi_2_Te_3_ [22,23] and can be expected to change in shape and magnitude based on material system.

**Figure 2:**
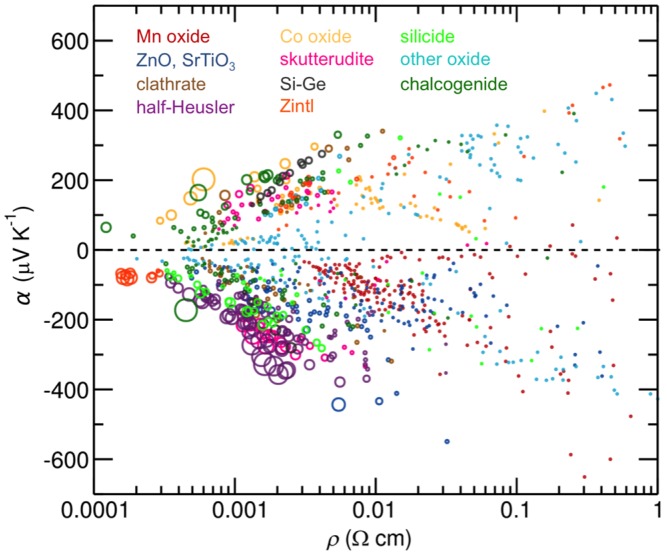
Transport properties of thermoelectric compounds reported to date, organized by class of compound. Marker radius is proportional to the reported power factor. Figure adapted from work by Gaultois *et al.* [26].

To find materials with even higher *zT* values, the community is challenged to find avenues to increase the power factor above that which can be obtained by optimizing the carrier concentration (Figure 1) [22,23]. There are several promising leads as to how this might be accomplished and the community is poised for a breakthrough that would lead to another increase in research activity. Large power factors require both large conductivities and simultaneously large Seebeck coefficients. While the search in the 1950s focused on narrow gap semiconductors, high performance thermoelectric materials generally have electrical resistivity values less than 1 × 10^−5^ Ωm at 300 K which is considerably more conductive than Mott predicted [24,25] for his minimum metallic conductivity. Compounds with metallic conductivity generally have low Seebeck coefficients, but high power factor compounds violate this principle. A recent review highlighted how infrequently metallic conductivity is found with large Seebeck coefficients as shown in Figure 2, adapted from work by Gaultois *et al.* [26]. High power factors result from interesting physics that make the compounds unusual. For bismuth and lead telluride compounds, a high band degeneracy perhaps with band asymmetry results in an unusually high Seebeck coefficient despite their metallic behavior [27]. Skutterudite structured compounds can have unusually high mobility values, leading to high conductivities at lower carrier concentrations [28,29]. Correlated electron and electron spin interactions in layered cobalt oxides, perhaps combined with an unusual band structure, results in very high Seebeck coefficients for a metallic compound [30,31,32]. High power factors are often found in compounds having correlated electron behavior, for example heavy Fermion materials [33,34] and compounds with large electron-phonon coupling often associated with charge or spin density wave behavior [35].

There are relatively few reports of taking a high performing material and enhancing the power factor and they generally fall into two categories. One successful approach has been to dope PbTe with either sodium or thallium in a manner that both controls the doping and alters the band structure [36,37]. The second strategy involves creating a nanocomposite, with material A present as nanoinclusions within a matrix of an already high performing thermoelectric material B. The nanoinclusions of A act both as a dopant and a scatterer of heat carrying phonons, but also have been suggested to increase the power factor either by scattering electrons of different energy at different rates or by doping in a manner that preserves high carrier mobility [38]. This second approach has become the focus of considerable effort, although the main contribution to enhanced *zT* still arises from a lowering of the lattice thermal conductivity through the scattering of heat carrying phonons.

### 2.4. Nanocomposites

A challenge in the efforts to optimize nanocomposites has been to find that part of the nanocomposite that is enhancing performance. Each of the constituents of the composite have their own band structures, and charge transfer will occur between them to lower the total energy. The surfaces of one or both constituents are likely to distort, changing the band structure from that of the bulk material. The charge transfer between the nanoinclusion and the matrix, similar to modulation doping in semiconductor superlattices, can control the carrier concentration without significant lowering of the carrier mobility due to impurity scattering [39]. The techniques available to characterize the nanostructure, including the local compositions and local structures at interfaces, are limited and provide only local views of what is likely to be a broad distribution of structural configurations at the interfaces. Understanding how to modify the structure at interfaces and tuning the Fermi level of the nanoinclusions via alloying to control doping remains a challenge. It offers opportunities to adjust parameters in ways not available to a single constituent system. An apprehension remains concerning the long-term stability of these nanoengineered systems under the high temperatures and temperature gradients during operation as part of a thermoelectric device. Hopefully a balance can be found that maintains the gains in performance while preserving the long-term stability and reliability of thermoelectric devices.

## 3. Misfit Layer Compounds

**Figure 3:**
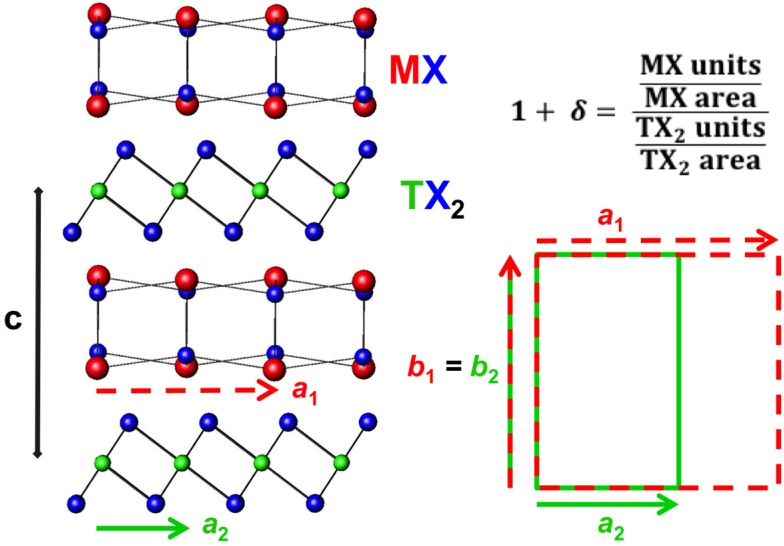
Structural summary of misfit layer compounds. General Superlattice structure (**left**) with in-plane lattice depiction (**bottom right**) and calculation of misfit parameter based on difference in in-plane packing density (**top right**).

Misfit Layer Compounds (MLCs) are an interesting class of precisely ordered thermodynamically stable nanocomposites and the discovery of high *zT* in [Ca_2_CoO_3_]_p_CoO_2_ resulted in examining chalcogenide compounds with similar structures. We will not discuss the oxide materials here, as very good overviews already exist [40,41]. The misfit layered chalcogenides are composed of a layered dichalcogenide and a rocksalt like structure as seen in Figure 3 with the general formula [(MX)_1+δ_]_m_(TX_2_)_n_, where M is Sn, Pb, Sb, Bi or a rare earth, T is Ti, V, Nb, Ta or Cr, and X is S or Se. There are several excellent reviews of these compounds and their unusual structures and properties [42,43,44,45]. They provide an interesting potential opportunity to study the effect of interface structure and local distortions on thermoelectric properties, because the precisely periodic structure enable the structure to be solved using higher dimensional crystallography to take into account the mismatch in in-plane area of the two constituents [46]. Unfortunately it is only possible to prepare compounds with small m and n due to synthetic limitations.

### 3.1. Structure

**Figure 4:**
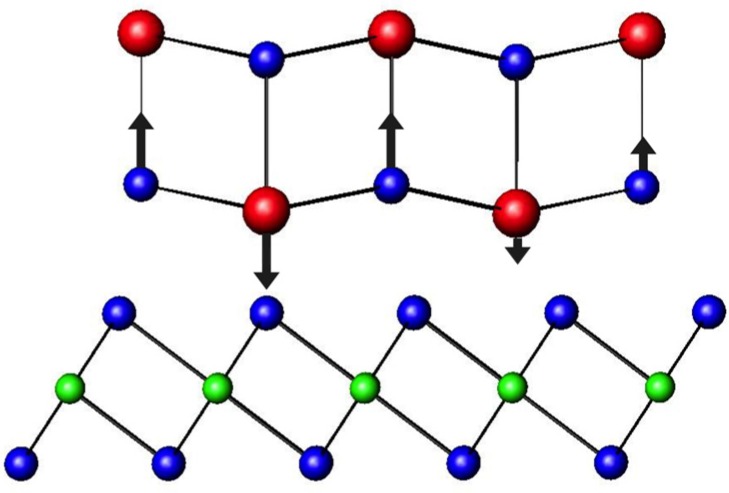
Two-dimensional representation of modulation in the *a*-direction and the resulting changes in local environment. The black arrows represent coulombic interactions expected based on proximity to X atoms in the adjacent TX_2_ layer.

Initial reports misidentified the misfit layer chalcogenide compounds as MTX_3_, until single crystal diffraction experiments showed the complex superlattice structure with two unique constituents, the history of which is nicely summarized by Wiegers [42]. The unit cell is generally defined with the *c*-axis normal to the constituent layers, which typically consists of a bilayer of rocksalt interleaved between dichalcogenide layers, with only van der Waals bonding thought to occur between the two structures [42,43,44,45]. Compounds reported to date have generally been discovered through high temperature synthesis routes, with most of the reported compounds having m = n = 1 or m = 1, n = 2. The relative displacement between the constituents, the distortions of each of the constituents discussed below, and different stacking arrangements of the dichalcogenide layers produce a considerable number of different unit cell symmetries as has been extensively reviewed by Wiegers in 1996 [42]. The two constituents display large distortions from the expected bulk structures, as the two lattices conform to one another typically yielding a commensurate *b* lattice parameter, as shown in Figure 3. The *a* lattice parameters, however, are typically not commensurate and result in a difference in the in-plane packing density of the two constituents, expressed by the 1 + δ term, and calculated as shown in Figure 3. The incommensurate structure between the distinct lattices results in a modulation function that reflects the changes in the local environment in the crystal structure [46]. A simple 2-D depiction of this phenomenon is given in Figure 4, where the proximity of M cations and X anions in the rock salt structured constituent with respect to the neighboring X atoms in the TX_2_ layer varies with position. The termination of the 3 dimensional rock salt structure causes a puckering distortion of the MX layer. Charge transfer between the constituents and local charges on different atoms result in electrostatic interactions that affect the puckering distortions in the MX layer. It should be noted that some compounds containing BiX and SbX, where X = S or Se, deviate from this basic structural motif [42]. The complexity of the crystal structure, the lack of distinct bonds between constituents, and the anharmonic potentials for atoms at the interfaces has led to suggestions that this structural motif could provide an ideal platform for a phonon-glass electron-crystal behavior, with potentially large *zT* values if the proper material system could be identified.

The stability of misfit layered compounds has long puzzled researchers [47,48,49,50,51,52,53,54]. The structure of misfit layer compounds has resulted in these compounds being discussed as two weakly interacting constituents with only van der Waals interactions at the interface. However, since these compounds are thermodynamically stable relative to a mixture of the constituent binary compounds, the enthalpy and/or the entropy of the misfit compound must result in a lower total free energy. These compounds have been proposed to be entropy stabilized through alloying between layers [52]. However, the entropy of a mix of two compounds is almost always higher than a single compound, unless there is extensive alloying. A second group of papers suggests that the total enthalpy of the misfit compound is lower than that found in the mix of the constituent binary compounds. The MX-TX_2_ interface therefore must have stronger bonding than the sum of the bonding between 00*l* planes in the MX constituent plus the van der Waals interaction between TX_2_ planes in the dichalcogenide. Based on the lack of charge transfer observed in photoelectron spectroscopy and band structure calculations, the interlayer bonding has been described as a covalent interaction [49,51]. Charge transfer between the two constituents, resulting in an ionic interaction between layers that stabilizes the compounds, is a third suggested stabilization mechanism. The amount of charge transfer is large for MX constituents where M is a rare earth and has been suggested to be small if M is a formally divalent cation such as Sn or Pb [42,48].

### 3.2. Thermal Transport Properties

Misfit compounds have been proposed as potentially ideal thermoelectric materials, with the dichalcogenide layer providing a region of high-mobility that provides the electron-crystal electronic structure, interwoven with the MX layer between the dichalcogenide layers acting as a phonon-glass by suppressing the transport of phonons by the structural mismatch between the MX layer and TX_2_ layer which disrupts the periodicity of dichalcogenide perpendicular to the layers [55]. It should be noted that these two interactions are orthogonal to one another in the structure. The first thermal conductivity study of a misfit layered chalcogenide that we could find a report of was done on (Yb_0.95_S)_1.24_NbS_2_ in 2004 in response to the high performance discovered in the structurally related layered cobalt oxides [56]. A very low total thermal conductivity of 0.80 WK^−1^m^−1^ was measured at 300 K. Subsequent measurements on (LaS)_1.20_CrS_2_ and (LaS)_1.14_NbS_2_ measured total thermal conductivity values between 1.2 and 1.5 WK^−1^m^−1^ [57]. Total thermal conductivity values of 2.8 WK^−1^m^−1^, 2.4 WK^−1^m^−1^ and 2.8 WK^−1^m^−1^ respectively have been reported by Koumoto for the misfit compounds containing two TiSe_2_ layers per unit cell, (BiS)_1.2_(TiS_2_)_2_, (SnS)_1.2_(TiS_2_)_2_ and (PbS)_1.18_(TiS_2_)_2_ [55,58]. The differences between the total thermal conductivities reported for these compounds are dominated by differences in the magnitude of the electrical contribution which varies with carrier concentration. The lattice thermal conductivity of these compounds range between 0.7 WK^−1^m^−1^ for (BiS)_1.2_(TiS_2_)_2_ to 1.2 WK^−1^m^−1^ for (PbS)_1.18_(TiS_2_)_2_. The extraction of these total thermal conductivities depends on the validity of the Wiedemann-Franz law, *k*_e_ = *L*_0_*T*σ and the assumed value for the Lorenz number *L*_0_ = 2.44 × 10^−8^ J^−2^C^−2^K^−2^.

### 3.3. Electrical Transport Properties

The transport properties of misfit layer compounds are generally thought to be dominated by the dichalcogenide layer, and can vary from semiconducting to metallic based on the transition metal and chalcogen, the number of *d*-electrons of the transition metal and the coordination environment [45]. Resistivity, Hall measurements, optical reflectivity and optical transmission experiments in (MS)_1+δ_TS_2_ have been used to understand the electronic interaction between the two constituents. Compounds with M = Pb, Sn and T = Ta, Nb display carrier concentrations close to that expected for the volume dilution effects expected based on the supercell [49,50,59,60,61], although carrier concentrations are typically about 10% lower. The lower than expected carrier concentrations have been used to support charge transfer between MS and TS_2_ [62] and also cation substitutions between the different constituents [47]. Compounds with M = Ce, La, Sm, Tb and T = Ta, Nb show a decrease in carrier concentration too large to result from volume dilution, which has been attributed to electrons transferred from the rocksalt to the *p*-type TaS_2_ and NbS_2_ layers [60,62,63].

The presence of the MX layers results in vastly different properties for MLCs relative to the bulk TX_2_ compounds with charge transfer from the MX constituent changing carrier concentrations from the bulk values of the parent dichalcogenide [45]. The distortion of the constituent lattices undoubtedly also affects the transport properties, particularly the mobility of the carriers when compared to the bulk dichalcogenide. If electrons are indeed transferred from the rocksalt to the dichalcogenide to fill lower energy states, this results in partially filled bands in both constituents, with the higher mobility carriers in the dichalcogenide layer responsible for the transport properties. This proposed mechanism of conduction explains the change in sign of both measured Hall and Seebeck coefficients for a number of misfit layered compounds when compared to the pristine bulk dichalcogenides and their intercalates [45,63,64,65,66]. This suggests that selection of the two proper constituents could provide a method of optimizing the carrier concentration to maximize the power factor. Such use of charge transfer between constituents is similar to modulation doping in semiconductor heterostructures first demonstrated in the late 1970s [67,68], where impurity atoms are added to a layer that is spatially separated from the conducting states in order to maintain high carrier mobility in the conducting layers while varying carrier concentration. Most of the studies in metallic Nb and Ta based sulfide compounds have been reported to have Seebeck coefficients less than |50| μVK^−1^ due to high carrier concentrations [42]. While it should be possible to control carrier concentration via doping of the rock salt structure, this has not been reported, presumably due to the difficulty of obtaining single phase products from the high temperature synthesis approaches commonly used.

Compounds based on TiS_2_ were reported to have lower carrier concentrations than the Nb and Ta based compounds, approximately 10^21^ cm^−3^ compared to 10^22^ cm^−3^, producing higher Seebeck coefficients (as high as |70| μVK^−1^) with still reasonably high conductivity values [55,66]. The group of Koumoto reported the synthesis of (MS)_1+δ_(TiS_2_)_2_, where n = 2 with M = Bi, Pb and Sn [55]. The addition of the extra layer of TiS_2_ per unit cell in the PbS and SnS resulted in nearly a factor of 2 increase in the Seebeck coefficient when compared to the n = 1 compounds. The Bi compound showed a marked increase in both carrier concentration and conductivity, presumably because it is donating its additional valence electron to the TiS_2_. (PbSe)_1.16_(TiSe_2_)_2_ reported by Giang and Cava is the only misfit layer based on TiSe_2_ with a reported Seebeck coefficient in the literature to date [65]. This compound has a lower electrical conductivity and comparable Seebeck coefficient to the sulfur analog prepared by Koumoto [55]. The difference in measurement techniques (single crystal *versus* pressed pellet) and the variability inherent in solid state synthesis makes it difficult to draw conclusions from the comparison. Combined with the thermal conductivity values discussed earlier, these TiX_2_ based compound reach *zT* values of ~0.3 at room temperature, similar to that reported for [(Yb_0.95_S)_1.24_]NbS_2_ [56]. It is important to note that none of these compounds have been optimized to have the carrier concentration that yields the highest power factor.

The anisotropic crystal structure of the misfit layer compounds also results in anisotropic electrical properties, although the magnitude of the anisotropy varies considerably in the literature. Wiegers reported that the cross plane electrical resistivity was four orders of magnitude higher than the in plane resistivity for TiS_2_ containing misfit layer compounds with SnS and PbS [66], which is probably an overestimate since the cross plane measurement was done using a two contact method which includes the contact resistances. Optical techniques have also been used to look at the anisotropy in electrical behavior to avoid contact resistance as an issue. The near-infrared reflectivity was reported to be dominated by free carrier plasma edges and suggests a much smaller anisotropy in resistivity than 10^4^ [60,63]. More recently Nader has reported measurements on single crystals of (SmS)_1.25_TiS_2_ and found the in plane resistivity a factor of 50 lower than the cross plane resistivity [69]. With all of the recent interest in heterostructures of different layered materials stacked in ordered arrangements with anticipated new properties arising from 2-dimensional behavior of the layers, more measurements of cross plane properties are needed.

The chemical flexibility of misfit layer compounds provides an interesting platform to explore structure-function relationships, to explore the phonon glass-electron crystal concept of Slack, and to understand phenomena of importance to improving performance of thermoelectric materials. The pseudo-independent band structures in these layered materials offer the opportunity to adjust one layer to affect the other through modulation doping. To date, the extent to which this class of compounds has been explored is limited by the constraints of thermodynamic stability at the high temperatures used in traditional solid-state chemistry synthesis approaches. Their potential flexibility remains relatively unexplored. The discovery of the layered cobalt oxide based compounds suggests that misfit type compounds, consisting of two unique constituent lattices, provide a flexible structural motif that has relatively low thermal conductivity and potential new avenues to improving the power factor [40,41]. Controlled substitution, alteration of layering schemes, and identification of constituent pairings that result in optimum materials properties remain to be explored.

## 4. Ferecrystals

**Figure 5:**
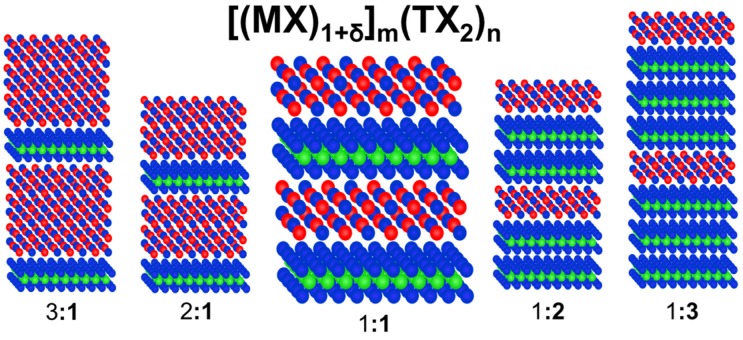
The thermodynamic stability of the reported Misfit Layer Compounds (MLCs) suggests that local free energy minima must exist for other values of m and n.

A thermodynamically stable (MX)_1+δ_TX_2_ misfit layer compound implies that the interface and interaction between the constituent layers must lower the free energy of the misfit layer compound below that of a mixture of the two binary compounds. The formation of an interface between the constituents results in a decrease in free energy, which explains the tendency to form misfit layer compounds with a single layer of each constituent using traditional high temperature synthesis approaches. Hence, it is straightforward to postulate that increasing the thickness of one or both of the constituent layers in the repeating unit, depicted in Figure 5, should yield a compound that is at least a local free energy minimum. The challenge is coming up with a kinetically controlled route to any specific compound within the very large set of potential compounds [(MX)_1+δ_]_m_(TX_2_)_n_. The synthesis approach needs to control the specific placement of interfaces in each unit cell and prevent the formation of additional interfaces as the system seeks to lower its free energy by forming the maximum number of interfaces.

### 4.1. Synthesis

A kinetically controlled synthesis approach has been developed over the past 25 years to access metastable compounds under conditions where they are kinetically trapped via the self-assembly of vacuum deposited amorphous precursors containing designed sequences of ultrathin modulated elemental layers [70,71,72,73,74]. In the last 10 years, it has been shown that precursors prepared with local composition profiles similar to that found in a misfit layer compound will self-assemble in a short time (minutes) at low temperatures (300–400 °C) because only short-range diffusion is required. By preparing designed sequences of binary layers, it has been possible to trap kinetically stable compounds with specific m and n values not obtainable through traditional methods [70,71,72,73,74]. This approach requires the precise calibration of the elemental layers to provide the correct composition of each of the precursor’s elemental bilayers corresponding to the constituent’s stoichiometry, the correct ratio of composition between the two constituents as defined by the misfit parameter, and the correct total amount of material per constituent layer (either M-X or T-X) to allow for the formation of a single structural repeating unit of each of the targeted constituents. This procedure has been previously described in considerable detail [72]. Typically precursors are prepared with ≤ 5% excess chalcogen to account for any losses during annealing. The synthesis of specific compounds in the system is done by repeating the deposition of the elemental M-X and T-X bilayers the desired number of times to achieve the targeted m and n values. Low temperature annealing self-assembles the precursors into the targeted kinetically stable intergrowth structures.

### 4.2. Structure

**Figure 6:**
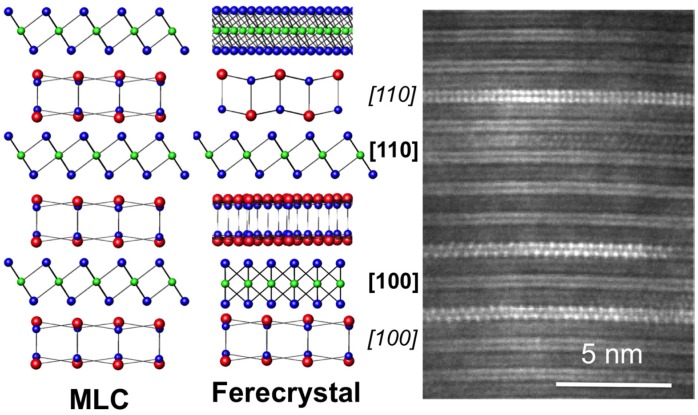
Comparison of 3-D crystals observed in misfit layer compounds and the turbostratic disorder observed in the metastable ferecrystals (**left**). HAADF-STEM image of ferecrystalline (SnSe)_1.2_TiSe_2_ showing turbostratically disordered layers (**right**). Zone-axes are visible that help identify the constituents.

This approach has been used to prepare metastable compounds of the general formula [(MX)_1+δ_]_m_(TX_2_)_n_ with M = Bi, La, Pb, or Sn; T = Ti, V, Cr, Mo, Nb, W, or Ta; and X = Se or Te that contain an integer number m bilayers of MX separating blocks of TX_2_ that are n X-T-X trilayers thick. The difference in the synthesis approach results in several important structural differences between the thermodynamic and metastable misfit layer compounds. First, the constituent structures do not distort to make a common in-plane lattice parameter resulting in independent in-plane diffraction patterns for the two constituents, and less strained constituent structures [70,71,72,73,74,75,76]. The metastable compounds also exhibit turbostratic disorder, or rotational disorder about the *c*-axis, which has been confirmed both locally via electron microscopy (Figure 6) and globally using area diffraction techniques [70,71,72,73,74,75,76]. Discrete *00l* and *hk0* reflections can be observed in area diffraction patterns, but the lack of 3-D periodicity results in streaking in the *l* direction for *hkl* reflections where (*h*, *k* ≠ 0; *l* ≠ 0) due to the lack of long range coherence between constituent layers. This disorder disrupts the 3-D crystallinity in the material, and these metastable compounds are referred to as ferecrystals, from the latin root *fere*, meaning almost. In some systems, there is evidence for longer-range order in the *c*-direction [77,78]. Presumably, the lattice mismatch and the resulting modulated alignment of MX dimers to the TX_2_ sheets plays an integral role in defining the energy landscape as a function of interlayer orientation. Work is underway to understand the observed local order and how to control the preferred interlayer orientation present in these systems.

**Figure 7:**
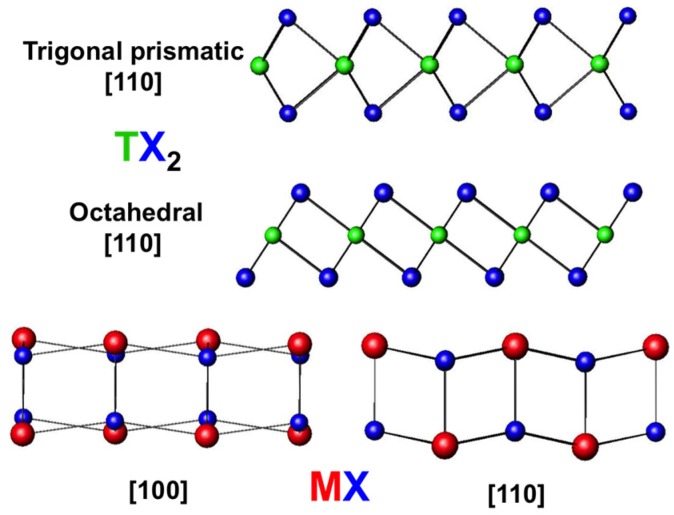
Structure types observed for TX_2_ layers. Compounds based on Ti and V are octahedrally coordinated, and those based on Ta and Nb are trigonal prismatically coordinated. The rocksalt like structure is also shown, with puckering distortions in the *c*-direction.

In most ferecrystal families, the TX_2_ structures can be fit to the bulk structure types. Systems containing Ta, Nb, Mo and W typically have trigonal prismatic coordination of the transition metal, while those containing Ti and V have octahedral coordination. A useful discussion of the structure of these binary compounds based on electron configuration was presented by Kertesz and Hoffman [79]. Zone axes for the expected structures (chevrons for trigonal prismatic and dumbbells for octahedral, see Figure 7) have been observed in electron microscopy studies of ferecrystals, and the in-plane structures have displayed reflections consistent with the bulk structures, with lattice parameters generally very close to those reported in bulk compounds [70,71,72,73,74,75,76].

Different stacking arrangements of TX_2_ layers result in a large number of polytypes in the bulk binary compounds [79,80]. Octahedrally coordinated systems such as TiX_2_ and VX_2_ form 1-T polymorphs, where the layers stack in an A-A repeating sequence, yielding a *c*-axis lattice parameter equal to the distance between transition metal atoms in two adjacent layers. Compounds containing Ta, Nb, Mo and W in trigonal prismatic and/or octahedral coordination form more complex stacking sequences by varying the synthesis conditions. Ferecrystal containing TiX_2_ and VX_2_ have each block of n trilayers forming as a 1-T polymorph, but the orientation of the 1T blocks varies between blocks. Ferecrystals containing Ta, Nb, Mo and W show mostly trigonal prismatic coordination and typically have a random stacking sequence both in and between dichalcogenide blocks [70,71,72,73,74,75,76,81].

The structure of the MX constituents in ferecrystals varies with the thickness of the block, as surface and volume free energies compete to create the lowest energy structure. PbSe layers adopt a square basal plane structure, but there is a pairing distortion with alternating long and short distances between the rock salt planes along the *c*-axis. This distortion decreases as m increases, and when m ~ 6 the structure appears to be bulk, with a puckering distortion of the surface layer [82]. SnSe is either square or displays a slight distortion from a square basal plane, at low values of m. At higher values of m, SnSe shifts towards the orthorhombic (GeS structure) for α-SnSe [73]. A more complete summary of the in-plane structures of ferecrystalline compounds and a comparison with the thermodynamic compounds is available elsewhere [75].

### 4.3. Thermal Conductivity

The lack of long-range order in ferecrystals prevents the formation of phonons, which are present in the crystalline misfit layer compounds. This results in incredibly low thermal conductivity, including the lowest lattice thermal conductivities ever measured for a full dense solid, on the order of 0.05–0.10 Wm^−1^K^−1^ along the *c*-axis [83,84]. The cross-plane thermal conductivity is the sum of the series thermal conductivity of the individual components. Along the constituent planes, the thermal conductivity is higher, 0.4–0.5 Wm^−1^K^−1^ measured on a number of free-standing films annealed under a variety of conditions [85]. These very low lattice thermal conductivities are very advantageous for potential thermoelectric applications, as it reduces the loss of efficiency due to heat flow through the module. Turbostratic disorder is currently the most effective strategy known to realize a “phonon glass”.

### 4.4. Transport Properties

The magnitude of the in-plane electrical resistivity of ferecrystals and misfit layer compounds with the same composition and nanoarchitecture are within a factor of 10 of one another [65,86]. The resistivities of the metastable compounds have very little temperature dependence when compared to their thermodynamic analogs. Interestingly, the low residual in-plane resistivity ratio is not due to an increased resistivity at low temperatures, which may be expected from impurity or other fixed defect scattering. For systems where both thermodynamic and metastable compounds are reported, the resistivity at low temperatures is similar in magnitude [86,87,88,89]. Misfit layer compounds with high carrier concentrations show behavior expected for a metal, with resistivity increasing linearly with temperature due to increased electron-phonon scattering. The metastable analogs, while showing similar magnitudes of resistivity below 50 K, do not show the increase at higher temperatures. This lack of temperature dependence has been attributed to the lack of electron-phonon scattering at higher temperatures [88,89]. In general, ferecrystals have a higher mobility than the misfit layer compounds. The lack of distortions in the TX_2_ constituents in the ferecrystals may play a role in the enhanced mobility observed. Improving mobility in materials is one way to improve the conductivity of a material, without significantly affecting the Seebeck coefficient.

### 4.5. Charge Transfer between Constituents

The ability to synthesize ferecrystalline compounds with a much broader range of m and n has provided a mechanism to systematically study constituent interaction. Two series of metallic compounds, [(PbSe)_1+δ_]_m_NbSe_2_ and [(SnSe)_1+γ_]_m_NbSe_2_, have systematically decreasing carrier concentration when normalized to the NbSe_2_ layer as m increases [87,89]. NbSe_2_ itself is a metal with a half filled band, and the Hall coefficient for both these series is positive and systematically increases as m increases. This suggests an increasing transfer of electrons from the rocksalt to the dichalcogenide as m increases. Work on TiSe_2_ containing ferecrystals also suggests charge transfer, with the m = 1, n = 2 compounds displaying similar trends to those observed for the Sn, Pb and Bi MLC analogs with TiS_2_. The BiS and BiSe containing MLCs and ferecrystals have a higher carrier concentration, consistent with the donation of more electrons to TiX_2_ per Bi atom, and hence a higher conductivity than the SnX and PbX compounds. This charge transfer between constituents might account for the stability of these compounds. Calculations of the energetic gain through coulombic interactions, which can be thought of as energy stored in an atomic scale capacitor, suggest that significant stabilization may be gained through charge transfer [77]. The varying level of charge transfer based on constituent suggests that adjustments to the rocksalt layer could be used to affect carrier concentration in the conducting dichalcogenide layers in a manner similar to modulation doping in III–V superlattice systems grown via molecular beam epitaxy [67,68].

### 4.6. Thermoelectric Potential

**Figure 8:**
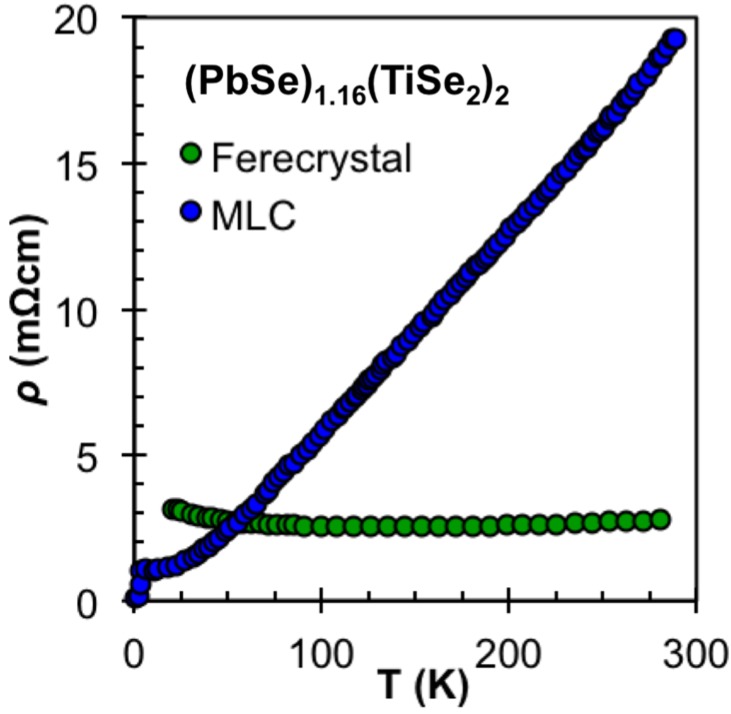
MLC [65] and ferecrystal (from set A in this work) temperature dependent in-plane resistivities. The turbostratically disordered compound is found to be almost temperature independent due to the disruption of phonons in the out of plane direction.

The extremely low lattice thermal conductivities of ferecrystals and the ability to tune their structures make them attractive test systems and potentially useful thermoelectric materials. Turbostratically disordered compounds containing NbSe_2_, TaSe_2_, and VSe_2_ as the dichalcogenide constituent layered with either PbSe or SnSe have metallic behavior with carrier concentrations too high to produce large Seebeck coefficients [72,87,89,90]. As with the misfit layered compounds, it would be interesting to explore the properties of these group V transition metal dichalcogenides with rare earth monochalcogenides. Turbostratically disordered compounds containing MoSe_2_ and WSe_2_ with either PbSe and SnSe can have very high Seebeck coefficients, but they also have resistivity values that are much too high, leading to low power factors [74,91]. As with the thermodynamically stable misfit compounds, however, materials based on TiSe_2_ have shown to produce carrier concentrations on the order of 10^21^ cm^−3^, with high Seebeck coefficients and in-plane resistivity values on the order of 10^−5^ Ωm. To date, (SnSe)_1.2_TiSe_2_, (PbSe)_1+δ_(TiSe_2_)_n_ (n = 1, 2) and (BiSe)_1.15_TiSe_2_ have all been reported [77,86,88,92]. For TiSe_2_ based compounds, the only direct comparison between a ferecrystal and a misfit compound is for [(PbSe)]_1.16_(TiSe_2_)_2_ [65,86]. Interestingly, the ferecrystal has an order of magnitude higher conductivity at room temperature as shown in Figure 8 and α that is twice as large as the misfit layer compound. Such an increase in Seebeck coefficient and conductivity in unison is rare, and results in a factor of approximately 30 increase in the power factor. Although no Hall data were reported for either compound, the simplest explanation is that the ferecrystal has a lower carrier concentration and a significantly higher mobility.

## 5. Synthesis and Properties of [(PbSe)_1+δ_]_m_(TiSe_2_)_n_ with m, n ≤ 3

**Figure 9:**
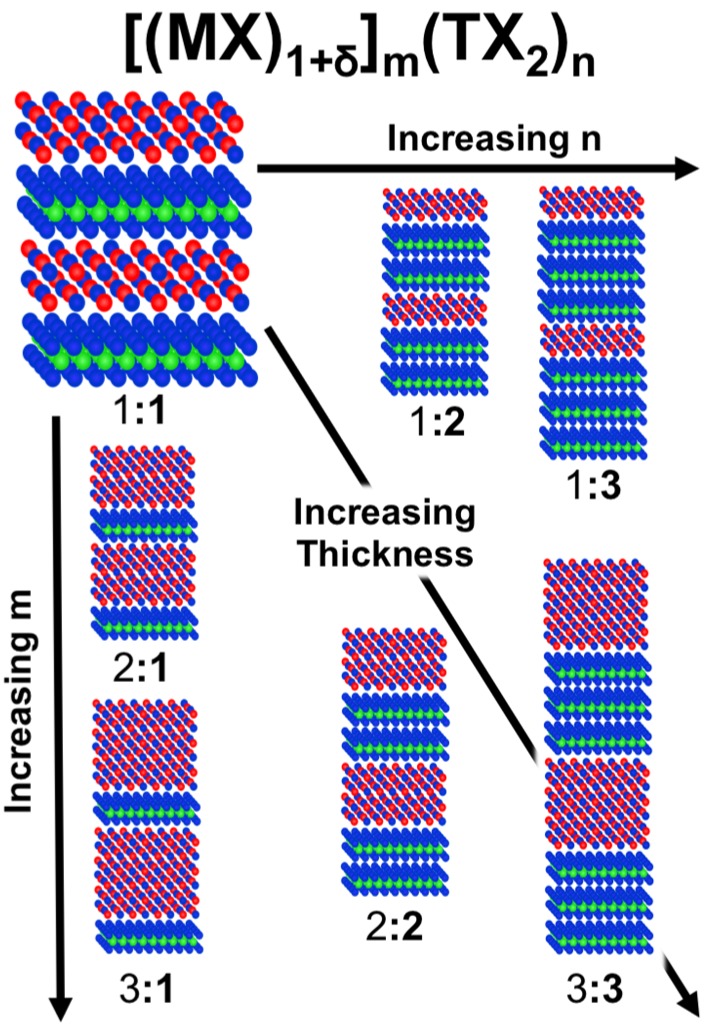
The ability to synthesize compounds with a variety of m and n values offers the opportunity to identify structure property relationships. Some of the first 10 compounds are given here with m, n ≤ 3.

The significant difference in properties between the ferecrystal and misfit compound [(PbSe)]_1.16_(TiSe_2_)_2_ lead us to investigate other members of this family of ferecrystalline compounds. Fortunately, the modulated elemental reactants synthetic technique provides an avenue to systematically prepare compounds with specific values of m and n. We decided to prepare all 10 compounds with m and n less than or equal to 3—*i.e.*, m:**n** = 1:**1**, 1:**2**, 1:**3**; 2:**1**, 2:**2**, 2:**3**; 3:**1**, 3:**2**, 3:**3** and the 1:**1**:2:**2** structural isomer of 3:**3**, with sets of compounds summarized in Figure 9. This sequence of compounds provides the opportunity to look for trends indicative of charge transfer between constituents. If the PbSe layer behaves as an electron donor and the TiSe_2_ layer as an acceptor, the carrier concentration for a series of constant m and increasing n should decrease, as the charge donated is spread over more layers.

### 5.1. Synthesis

**Figure 10:**
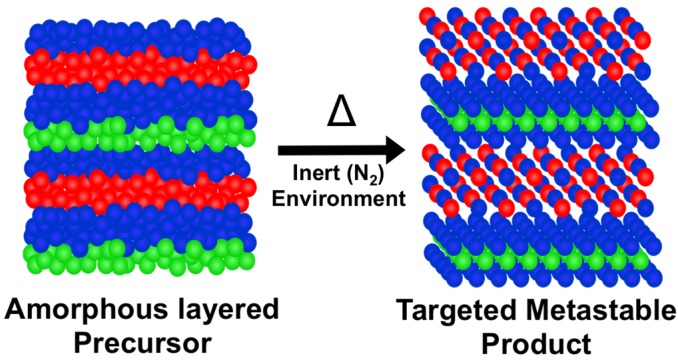
Modulated elemental reactant synthesis. Control of local composition and structure allows for the self-assembly of amorphous precursors into targeted metastable products.

The modulated elemental reactant synthesis approach is based on preparing a precursor that has local composition and overall nanoarchitecture similar to that of the desired final product. Gently annealing this precursor results in its self-assembly into the desired product as shown in Figure 10 for a 1:**1** compound. Depositing two layers of the Ti:Se layer instead of one layer as depicted in Figure 10 would result in the self-assembly of the 1:**2** compound. The calibration of the deposition system to prepare the precursors involves three steps. First, the ratio of the elements in the Pb:Se and Ti:Se layers have to be adjusted to correspond to the stoichiometry of the PbSe and TiSe_2_ layers desired in the final product. Second, the ratio of Pb to Ti has to be made to correspond to the misfit parameter, 1.16. Third, the absolute thickness of the Pb:Se and Ti:Se layers has to be adjusted while maintaining relative compositions such that the number of atoms in each layer corresponds to the structural unit that will self-assemble: a bilayer of PbSe and a Se-Ti-Se trilayer, respectively. A systematic procedure for accomplishing this using XRR and electron probe microanalysis [93] to determine thickness and composition respectively has been described in detail previously [72].

### 5.2. Structure

**Figure 11:**
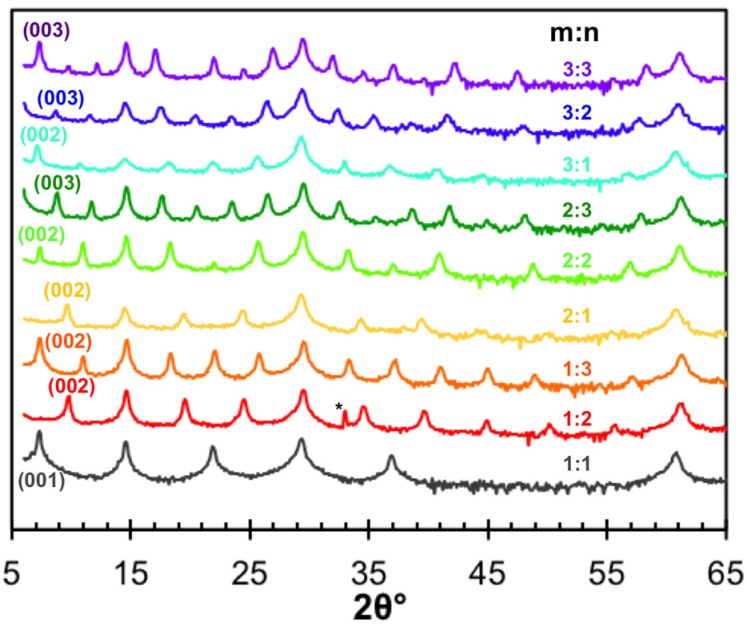
Diffraction patterns for the unique combinations of m:n (Cu Kα). All maxima can be indexed to *00l* reflections (* = substrate peak).

Figure 11 contains the diffraction patterns obtained from the 9 compounds made with a simple A:**B** structure. All of the reflections can be indexed as *00l* reflections of the targeted structures and the *c*-axis lattice parameters for each compound are given in Table 1. Several of the compounds were made several times, and the table contains the lattice parameters for each attempt. For each of the three series of compounds, 1:**n**; 2:**n** and 3:**n**, there is a systematic increase in the *c*-axis lattice parameter of 0.60(1) nm, corresponding to the thickness of the added Se-Ti-Se trilayer. This value is consistent with that reported for the binary compound TiSe_2_, 0.6004 nm and that found in the known [(PbSe)]_1.16_(TiSe_2_)_2_ misfit layered compound [65,94]. The systematic increase of the *c*-lattice parameter as m is increased by 1 is 0.62(1) nm, which is also consistent with that reported for the binary compound PbSe and the estimated thickness of PbSe layers in known misfit compounds [65,86,95].

**Table 1:** *C*-lattice parameter, electrical resistivity (ρ) and Seebeck coefficient (α) for compounds in the [(PbSe)_1+δ_]_m_(TiSe_2_)_n_ family synthesized for this study. The samples are organized by vacuum cycle in deposition equipment (black = set A, green = set B, blue = set C).

		c-lattice	*ρ*	*α*
m : **n**		(nm)	(10^−5^ Ωm)	(μV/K)
1 : **1**		1.218(1)	1.6	-
		1.2183(5)	2.7	−69
		1.218(1)	1.5	−57
1 : **2**		1.817(4)	1.3	-
		1.822(5)	3.7	−92
		1.817(1)	1.7	−79
1 : **3**		2.418(7)	1.1	-
		2.429(5)	4.3	−100
2 : **1**		1.832(4)	5.2	−68
		1.829(5)	1.6	−49
2 : **2**		2.430(2)	2.1	-
		2.432(3)	3.9	−89
		2.421(5)	3.3	−78
2 : **3**		3.033(4)	2.9	−103
3 : **1**		2.455(8)	1.5	−67
3 : **2**		3.091(7)	4.5	−94
3 : **3**		3.636(7)	2.2	-
		3.637(9)	4.3	−102
		3.637(6)	2.4	−85
1:**1:**2:**2**		3.643(4)	3.8	−74

**Figure 12:**
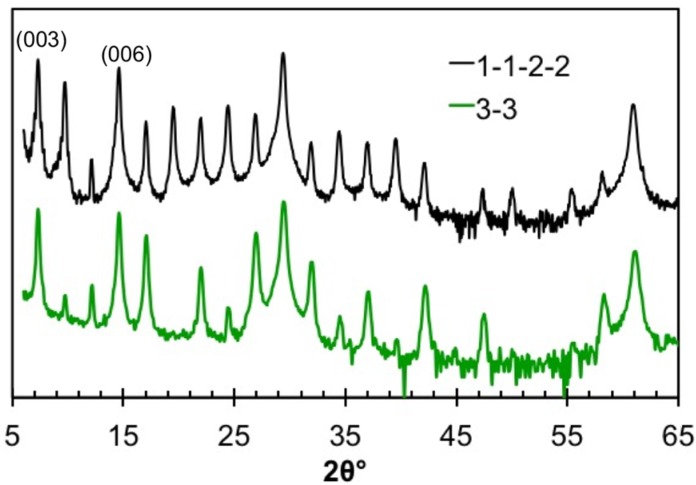
*00l* Diffraction pattern for the 3:**3** compound and its structural isomer 1:**1**:2:**2**. Differences in layering scheme result in changes to the relative intensity of diffraction maxima.

Figure 12 contains the diffraction patterns of the two structural isomers, 3:**3** and 1:**1**:2:**2**. As can be seen, the *00l* reflections are at the same angles, reflecting the similar *c*-axis lattice parameters of these compounds. The intensities of the reflections differ from one another as expected, because of the difference in the modulation of their electron densities along the *c*-axis. The lattice parameter of the 1:**1**:2:**2** isomer is slightly larger due to the additional interface between PbSe and TiSe_2_ in the unit cell.

**Figure 13:**
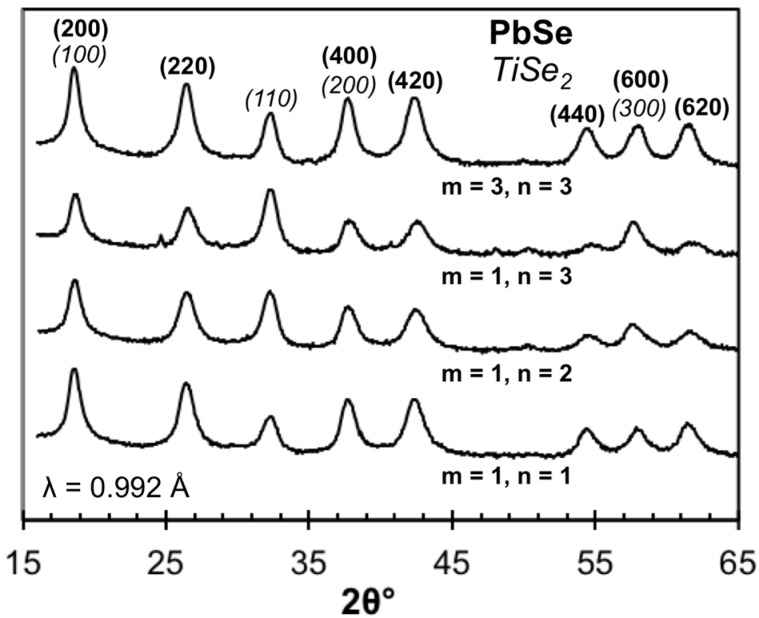
In-plane diffraction patterns for 1:**n** and 3:**3** compounds. Only *hk0* reflections for the two constituent structures are observed (labeled on the 3:**3** scan). The relative intensity of peaks change as expected for the varying ratio of constituent layers.

To get more information about the structure of these compounds, in plane diffraction scans were collected on a subset of samples and are shown in Figure 13. Only *hk0* reflections are observed due to the crystallographic alignment of the compounds with the substrate, forming a “2-D” powder along the in-plane direction. The reflections can be indexed as independent patterns of PbSe and TiSe_2_. The position of the reflections and their linewidths are similar in all 4 compounds, indicating that the in-plane lattice parameters do not change significantly as m and n are varied and that the PbSe lattice remains square and the TiSe_2_ lattice remains hexagonal in the basal plane as m and n are varied. The PbSe *a*-axis lattice parameter was found to be between 0.6109(4) nm and 0.6140(1) nm for these compounds. The TiSe_2_ *a*-axis lattice parameter was found to be between 0.3561(6) nm and 0.357(1) nm. These values are similar to those reported previously for PbSe-TiSe_2_ ferecrystals and misfit layer compounds [65,86,92].

**Figure 14:**
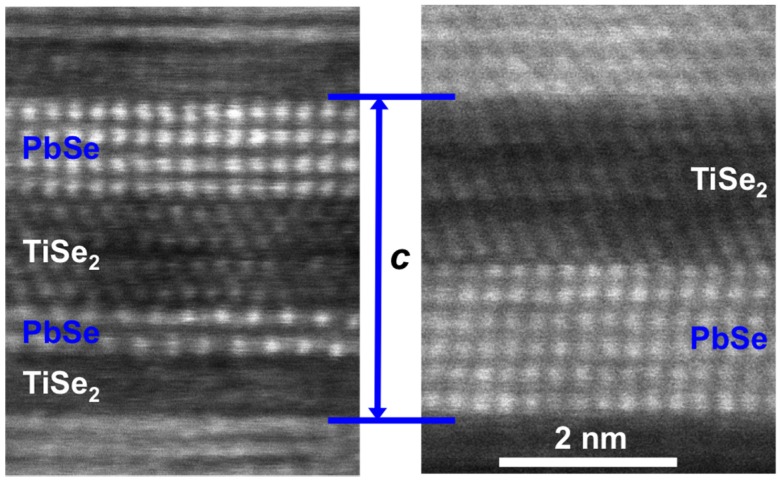
HAADF-STEM data for the 3:**3** and its structural isomer 1:**1**:2:**2**. The expected repeating units are observed, and turbostratic disorder is clearly visible in both compounds.

HAADF-STEM images were obtained on a subset of samples prepared for this study, and Figure 14 contains a close up image of the 3:**3** and 1:**1**:2:**2** isomers. Both compounds have unit cells containing 3 structural units of each constituent, but a different sequence of layers resulting in an additional interface in the 1:**1**:2:**2** isomer. The structures of the different layers can be seen to be different and different layers of PbSe and TiSe_2_ can be seen to have different orientations. However, within one block of each constituent the orientation remains the same. As observed in [(PbSe)_1+y_]_m_[MoSe_2_]_n_ compounds, the PbSe layer consists of pairs of planes, separated by slightly larger distances [82]. This distortion is thought to result from a competition between surface and volume free energy, with the observed distortion being the lowest energy state. The overall structure is consistent with that expected from the observed diffraction patterns and the designed nanoarchitecture of the precursors.

### 5.3. Electrical Transport Properties

**Figure 15:**
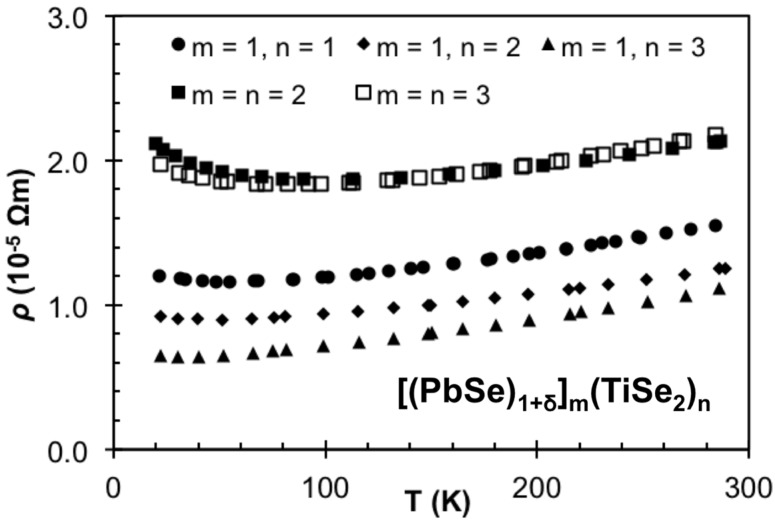
Temperature dependent in-plane resistivity for compounds from set A (black text in Table 1).

The temperature dependent in-plane resistivity for the samples in black in Table 1, which were all made in the same period of time with the same chamber calibration, are shown in Figure 15. The resistivity increases as m increases in the series 1:**n** and surprisingly the 2:**2** and 3:**3** compounds have higher resistivity than the 1:**1** compound. All compounds show the same basic temperature dependent behavior, with resistivity decreasing very slightly as temperature is decreased before either leveling out or slightly increasing at temperatures below 50 K. The two samples with higher resistivities also show a larger upturn at lower temperatures from room temperature. The lack of temperature dependence is consistent with prior reports of the resistivity of ferecrystals, and has been attributed to the turbostratic disorder which prevents organized vibrations with an out of plane component. The slight upturn at low temperatures has been attributed to electron-electron interactions leading to localization of carriers [96]. Another factor which will affect the conductivity is the magnitude of the anisotropy. Anisotropies of *σ*_ab_/*σ*_c_ ≈ 50 or more have been reported for misfit layer compounds, but this difficult measurement has not been done on any ferecrystal. The turbostratic disorder is likely to impact the magnitude of the anisotropy, and a very large anisotropy would suggest that 2-D effects might be causing the upturn in resistivity. The magnitude of the resistivity depends on both the number of charge carriers and their mobility through the lattice, both of which can be expected to change between compounds and due to slightly different compositions, between samples prepared in different deposition cycles. Slight changes in the composition would be expected to affect both the carrier concentration and carrier mobility, as impurity scattering and grain sizes change.

**Figure 16:**
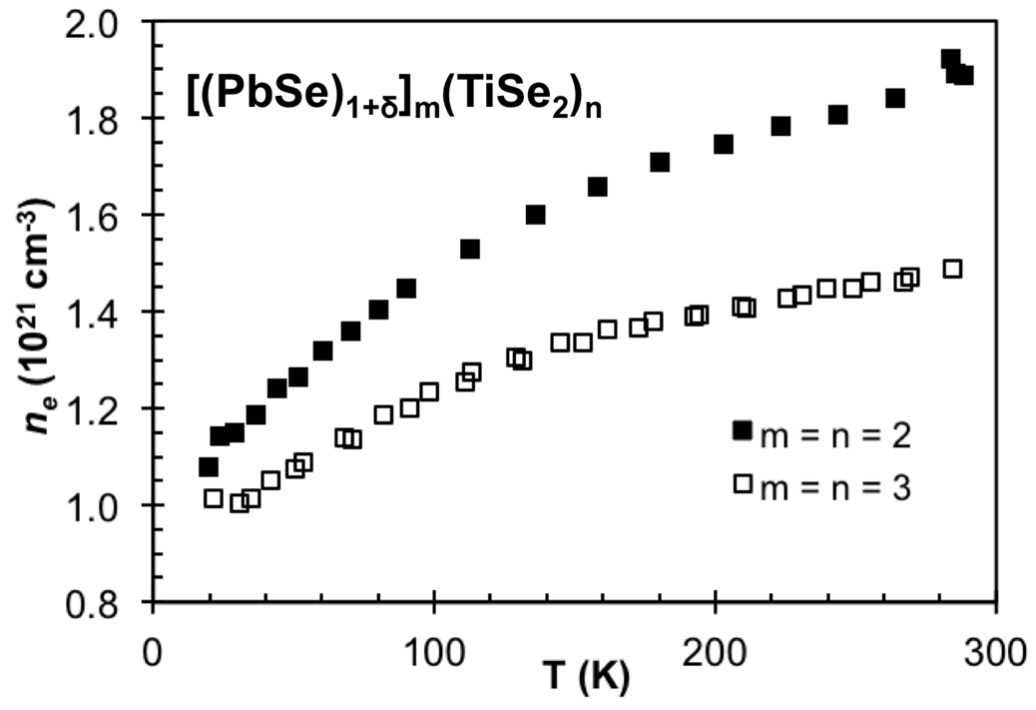
Temperature dependent carrier concentration (n_e_) for the m = n = 2 and 3 compounds, suggesting that the upturn in resistivity is in part due to a decrease in carriers.

**Figure 17:**
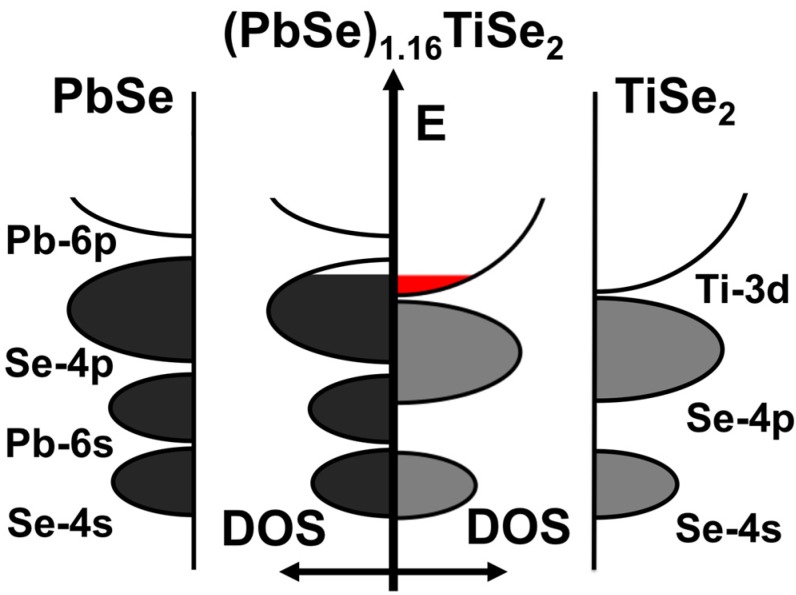
Schematic density of states diagram for (PbSe)_1.16_TiSe_2_ and the parent compounds. All evidence presented to date supports the transfer of electrons from the MX layer to the TiX_2_ layer, where conduction occurs. The transferred conduction electrons are shown in red.

In order to determine if carrier concentration or mobility differences are responsible for the different resistivity values of the compounds in Figure 15, Hall measurements were conducted and carrier concentration and Hall mobility were calculated assuming a single band model. For all samples the Hall coefficient was negative, indicating that electrons are the predominant charge carriers. This is in agreement with the prior work on TiS_2_ containing misfit compounds, where the TiS_2_ was assumed to be the predominant conducting layer and charge donation from the MS constituent resulted in the observed carrier concentration [55,66]. The room temperature results are summarized in Table 2. The carrier concentration systematically decreases as n is increased, and compounds with the same TiSe_2_ thickness but different PbSe thickness have similar carrier concentrations. The systematic decrease in carrier concentration with increasing thickness of TiSe_2_ is consistent with charge transfer from the PbSe to TiSe_2_, with the same amount of charge being diluted across more TiSe_2_ layers. The lack of a change in carrier concentration as m increases is more difficult to understand. The explanation is probably related to the structural distortions of the PbSe seen in the STEM images, which will change the band structure and perhaps leading to less charge transfer per layer as the number of PbSe layers increase. The mobility systematically increases in the 1:**n** compounds, varying inversely with the carrier concentration. The mobility remains relatively consistent within the m = n class of compounds. Figure 16 contains the temperature dependence of the carrier concentration for the 2:**2** and 3:**3** compounds, which indicates that the upturn in the resistivity is a result of a decrease in carrier concentration. This variation of the carrier concentration might also be a result of assuming a single band model. If there is charge transfer, between the constituent structures, two bands contribute to the conductivity with electrons in the TiSe_2_ layer having higher mobility than holes in the PbSe layer. Figure 17 contains a schematic density of states for the PbSe-TiSe2 system analogous to that proposed previously for the TiX_2_ based systems [42]. Changes in the position of the bands with temperature would cause the variation in carrier concentration. Differences in the temperature dependence of the mobility would also result in changes to both resistivity and Hall coefficients. Differences in the in-plane thermal expansion coefficients of the two different sublattices would change the bands for the constituents, leading to changes in carrier concentration. It is also possible that the observed changes result from more exotic phenomena, for example a crossover from 3 dimensional transport to 2 dimensional confinement. Experiments are in progress to test these speculative ideas. 

**Table 2:** Carrier concentration and mobility for compounds in set A. Carrier concentrations calculated from the Hall coefficient suggest that charge transfer is occurring between constituents.

m	n	*n_e_* (10^21^ cm^−3^)	*μ_e_* (cm^2^V^−1^s^−1^)
1	**1**	2.3	1.7
1	**2**	1.9	2.6
1	**3**	1.6	3.5
2	**2**	1.9	1.5
3	**3**	1.5	2.0

**Figure 18:**
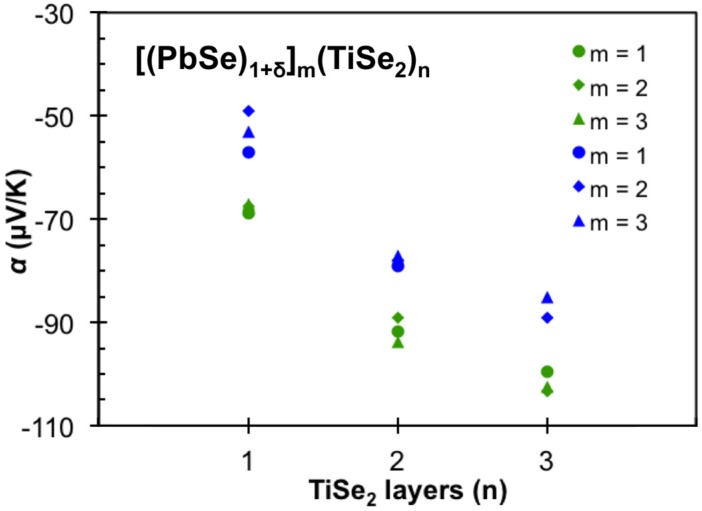
Seebeck coefficient (α) as a function of number of TiSe_2_ layers (n). The magnitude of α is seen to increase with n, independent of m.

Figure 18 shows the variation of the room temperature Seebeck coefficient as a function of the number of TiSe_2_ layers in the compounds for two sets of samples prepared in different deposition cycles. For all samples the Seebeck coefficient is negative, implying that electrons are the predominant carriers in agreement with the Hall measurements. The Seebeck coefficient is seen to increase in absolute magnitude as the number of TiSe_2_ layers is increased but changes only slightly in each set of samples as m is varied. This is consistent with the changes in carrier concentration calculated from the measured Hall coefficients. There is a larger difference between the two sets of samples prepared in different deposition cycles. This is consistent with prior investigations of samples prepared via self-assembly in our group, where samples synthesized within a given deposition cycle are relatively consistent and there is more variation between samples prepared in different deposition cycles. The variation between cycles likely arises from slight changes to composition and thickness of the precursor layers that result in differences in the type and density of defects. These subtle changes in defect density have proven difficult to quantify through structural characterization techniques, with nearly all the samples having very similar diffraction patterns. Differences in defect densities have been observed via electron microscopy studies, but the small areas investigated make it difficult to say that these observations are representative. Table 1 contains a summary of all of the samples investigated color coded by the deposition cycle in which they were prepared.

Table 1 also contains room temperature resistivity and Seebeck coefficients for the 3:**3** and 1:**1**:2:**2** sample. The resistvity of the 3:**3** compound was found to be slightly higher than that of the 1:**1**:2:**2** isomer, 4.3 × 10^−5^ Ωm compared to 3.8 × 10^−5^ Ωm. The Seebeck coefficient for the 3:**3** compound was −102 μV/K while that for the 1:**1**:2:**2** compound was found to be −74 μV/K, respectively. This is consistent with the discussion of the transport data for the other compounds, where thicker blocks of TiSe_2_ have lower carrier concentration, higher mobility and higher Seebeck coefficients. The data for the isomers, along with the other compounds with m, n ≤ 3 suggests that increased numbers of adjacent TiSe_2_ layers might be an avenue to increase the power factor. Additional studies on structural isomers, especially where more isomers are possible such as the 6 possible isomers containing 4 structural units of each constituent, might provide more insights to the role of the interfaces in these compounds.

## 6. Conclusions and Outlook

Misfit layer and similar compounds may provide a useful platform for the creation of high performance thermoelectric materials due to their low lattice thermal conductivity and potential for modulation doping on a sub-nanometer length scale. Like other material approaches to thermoelectric applications, understanding materials properties on a deeper level requires understanding reproducibility and the underlying effects on observed properties. In the case of bulk materials synthesized at high temperatures, it is generally assumed that thermodynamic equilibrium is achieved and can be reproduced. Metastable compounds represent an opportunity to test materials properties over a wider range of composition and structure. Improving control in the preparation of the precursors to ferecrystals would improve reproducibility.

The preferred orientation found in ferecrystals made via designed precursors makes structural characterization possible at a much more detailed level than a random mixture of a material in a matrix, The ability to synthesize these metastable variants of misfit layer compounds offers the opportunity to probe constituent interaction on a deeper level through systematically changing the thickness or composition of constituent blocks and identifying trends in the resulting transport properties.

The synthetic control offers the opportunity to make new compounds, providing the chance to observe trends in material behavior and potentially discovering properties not achievable through traditional approaches. The data presented on the [(PbSe)_1+δ_]_m_(TiSe_2_)_n_ family (m, n ≤ 3) compounds suggests that the relative energies of the valence and conduction bands of the two constituents is important to obtain materials with only a single carrier type. By making one of the constituents a wide band gap semiconductor such that the Fermi level of the other constituent winds up in the band gap, it might be possible to realize two-dimensional confinement. By doping the wide band gap semiconductor *n* type or *p* type, it should be possible to modulation dope the other constituent, either adding or removing carriers respectively.
